# Multiplex Serologic Assessment of Schistosomiasis in Western Kenya: Antibody Responses in Preschool Aged Children as a Measure of Reduced Transmission

**DOI:** 10.4269/ajtmh.16-0665

**Published:** 2017-06-07

**Authors:** Kimberly Y. Won, Henry M. Kanyi, Faith M. Mwende, Ryan E. Wiegand, E. Brook Goodhew, Jeffrey W. Priest, Yeuk-Mui Lee, Sammy M. Njenga, W. Evan Secor, Patrick J. Lammie, Maurice R. Odiere

**Affiliations:** 1 Division of Parasitic Diseases and Malaria, Centers for Disease Control and Prevention, Atlanta, Georgia; 2 Eastern and Southern Africa Centre of International Parasite Control, Kenya Medical Research Institute, Nairobi, Kenya; 3 Division of Foodborne, Waterborne and Environmental Diseases, Centers for Disease Control and Prevention, Atlanta, Georgia; 4 Center for Global Health Research, Kenya Medical Research Institute, Kisumu, Kenya

## Abstract

Currently, impact of schistosomiasis control programs in *Schistosoma mansoni*–endemic areas is monitored primarily by assessment of parasitologic indicators only. Our study was conducted to evaluate the use of antibody responses as a way to measure the impact of schistosomiasis control programs. A total of 3,612 serum samples collected at three time points from children 1–5 years of age were tested for antibody responses to two schistosome antigens (soluble egg antigen [SEA] and Sm25) by multiplex bead assay. The overall prevalence of antibody responses to SEA was high at baseline (50.0%). After one round of mass drug administration (MDA), there was minimal change in odds of SEA positivity (odds ratio [OR] = 1.02, confidence interval [CI] = 0.79–1.32, *P* = 0.89). However, after two rounds of treatment, there was a slight decrease in odds of SEA positivity (OR = 0.80, CI = 0.63–1.02, *P* = 0.08). In contrast to the SEA results, prevalence of antibody responses to Sm25 was lowest at baseline (14.1%) and higher in years 2 (19.8%) and 3 (18.4%). After one round of MDA, odds of Sm25 positivity increased significantly (OR = 1.51, CI = 1.14–2.02, *P* = 0.005) and remained significantly higher than baseline after two rounds of MDA (OR = 1.37, CI = 1.07–1.76, *P* = 0.01). There was a significant decrease in the proportion of 1-year-olds with positive SEA responses from 33.1% in year 1 to 13.2% in year 3 and a corresponding decrease in the odds (OR = 3.25, CI = 1.75–6.08, *P* < 0.001). These results provide preliminary evidence that schistosomiasis program impact can be monitored using serologic responses.

## Introduction

Schistosomiasis, caused by infection with *Schistosoma* spp., affects more than 200 million people worldwide.^1^ Prevalence and intensity of infection with *Schistosoma mansoni* peak between 10 and 15 years of age and gradually decline with age. In children, chronic schistosomiasis is associated with anemia and malnutrition and can compromise growth and cognitive development.^2^ Because of the influence school-aged children (SAC) have on transmission of schistosomiasis, mass treatment of this age group with praziquantel (PZQ) has been the cornerstone of schistosomiasis control activities.^3^ Until recently, disease burden and morbidity among preschool-aged children (PSAC) have remained understudied. However, recent research has shown that first infection is often acquired at a very young age,^4^–^8^ and there is growing evidence that the burden of disease among PSAC may warrant global attention. Although schistosomiasis-associated morbidity among PSAC is still not well defined, documented effects include fecal occult bleeding,^9^,^10^ anemia,^11^,^12^ and ultrasound abnormalities^13^; however, discriminating these symptoms from other potential infectious causes remains a challenge. Despite mounting evidence for the need, PSAC are not routinely screened or included in schistosomiasis mass treatment programs in large part due to the need for better diagnostic tools and the lack of a pediatric formulation of PZQ.

Currently, program impact in *S. mansoni*-endemic areas is monitored primarily by assessment of parasitologic indicators only. This is traditionally done by monitoring changes in prevalence and intensity of infection using the Kato-Katz stool examination method,^14^ which has long been the primary diagnostic tool used for *S. mansoni* and soil-transmitted helminth (STH) control programs. Although this method allows for relatively simple assessment of prevalence and intensity of infection, there are known limitations with its use. Logistical challenges are introduced with the short time needed to collect and process samples, and quality results are dependent on trained microscopists who can correctly identify eggs. Furthermore, as prevalence of *S. mansoni* and STH infection decreases, the sensitivity of the Kato-Katz often decreases in parallel.^15^,^16^ Recent development of a urine-based point of care circulating cathodic antigen test (POC-CCA) for *S. mansoni* has addressed some of the limitations with the Kato-Katz. A number of studies have compared the POC-CCA to Kato-Katz and found that it is more sensitive than the traditional stool-based test.^17^,^18^ However, there are still some questions about the specificity of the test, especially in low-prevalence settings.^19^ Although there has been significant emphasis placed on using stool- and urine-based diagnostic tools to monitor the impact of treatment programs, less emphasis has been placed on the utility of antibody detection tools as a way to measure impact. Reduced transmission of schistosomiasis can be assessed, in principle, by documenting a lower prevalence of infection-specific antibody. Although there may be limitations to using antibody responses among older age groups, documenting reduced infection incidence among cohorts of young children can be one of the most powerful measures of program impact. However, this measure has not been incorporated into most monitoring and evaluation strategies. Newly developed multiplex bead assays (MBAs) to detect antibodies against multiple antigens could make it possible to monitor the effect of treatment on infections, and these assays could potentially be used as an additional measure of program impact.^20^ Our study was conducted to evaluate the use of antibody responses as a way to measure the impact of schistosomiasis control programs.

## Methods

### Study site.

The study was conducted from 2012 to 2014 in Mbita subcounty, which borders Lake Victoria in western Kenya. The majority of residents are subsistence farmers, although fishing is the main commercial activity in villages near the lake. In addition to fishing, the lake is used for other occupations such as car washing and sand harvesting, and daily activities such as washing clothes and bathing. High rates of *S. mansoni* infection and malaria have been documented in the area.^21^,^22^ Before the start of the study, malaria interventions had been in place for several years, but no mass drug administration (MDA) for schistosomiasis had been conducted. A single round of MDA for STH infections had been conducted in 2009 for SAC by the Kenya National school-based deworming program.

### Study design.

The study was part of a multi-country project designed to evaluate the impact of integrated neglected tropical disease (NTD) control programs. In Mbita, SAC from schools within 5 km of Lake Victoria were screened to identify communities with *S. mansoni* prevalence ≥ 25%.^22^ Thirty villages that met the selection criteria were randomized into two study arms to compare different MDA strategies for schistosomiasis and STH programs. Fifteen villages were randomized to a community-wide treatment arm and the remaining 15 villages were randomized to a school-based treatment arm. In each of the 30 study villages, we aimed to enroll 100 PSAC (1–5 years) and their mothers or guardians. In addition, we aimed to enroll 100 individuals ≥ 6 years (with no upper age limit) to give us a total target sample size of 300 individuals per study village. In both study arms, parasitologic and serologic indicators were monitored at baseline (year 1) and annually following treatment. All monitoring was done in cross-sectional surveys in the selected villages.

### Ethical considerations.

The study was approved by the Scientific Steering and Ethics Review Committees of the Kenya Medical Research Institute (KEMRI, SSC number 2185) and of the Institutional Review Board of the U.S. Centers for Disease Control and Prevention (protocol number 6249) through a reliance agreement with KEMRI. The study was explained to potential participants and written informed consent was obtained from persons who agreed to participate. Parents or guardians provided consent for children < 18 years of age. In addition, children between 7 and 17 years were asked to provide verbal assent for their participation. All identifiable information was kept confidential and maintained by using a secure database with access restricted to essential study personnel.

### Data collection.

All study villages were visited between May and July of each study year. Community leaders were sensitized to the study details at least 1 week before the arrival of the field teams. On the day of sample collection, residents of the community were asked to come to a central location within the village. The study was explained, and potential participants were given an opportunity to ask questions. After obtaining informed consent, participants were assigned a unique identifier and asked to provide basic demographic information such as age and sex. In addition, information about the length of residence within the study village and bednet usage was collected. A single global positioning system coordinate per village was recorded at the site of data collection. All data were collected on smartphones (Motorola Milestone XT720, Motorola, Chicago, IL) through a modified version of the OpenDataKit application and uploaded to a secure SQL server.

### Stool and urine collection and diagnostic tests.

For each participant, an attempt was made to collect a single stool sample to be processed by the Kato-Katz method. Two slides were prepared from each stool sample, read independently by trained microscopists and examined for the presence of *S. mansoni* and STH (*Ascaris lumbricoides*, *Trichuris trichiura*, hookworm) eggs. Arithmetic means of the results from the duplicate slides were calculated and expressed as eggs per gram (EPG) of stool. Results for *S. mansoni* were categorized as light- (1–99 EPG), moderate- (100–399 EPG), and heavy- (≥ 400 EPG) intensity infections according to the current World Health Organization (WHO) thresholds.^3^ A single urine sample was collected and tested by dipstick (URiSCAN, YD Diagnostics in year 1 and Hemastix, Siemens Healthcare Diagnostics in years 2 and 3) to assess hematuria as a proxy for *Schistosoma haematobium* infection. In years 2 and 3, urine samples positive for hematuria were filtered and examined for the presence of *S. haematobium* eggs.

### Blood collection and diagnostic tests.

Blood was collected via a single fingerstick. To assess anemia, hemoglobin levels were measured using a portable, battery-operated hemoglobinomteter (HemoCue, Angelholm, Sweden) according to the manufacturer's specifications. Anemia was defined according to the Kenyan clinical guidelines: < 10.0 g/dL for children < 5 years old, < 11.0 g/dL for children 5–8 years old, and < 12.0 g/dL for individuals ≥ 9 years old; anemia was categorized as mild if hemoglobin was > 8.0 g/dL, moderate if 5.0–8.0 g/dL, and severe if < 5.0 g/dL^23^ after adjusting for altitude.^24^ Malaria infection status was determined by preparing thick blood films and using standard Giemsa staining techniques. Slides were examined by trained microscopists to determine malaria parasitemia, and positive infection was defined by the presence of one or more malaria parasites in 300 high-powered fields. Approximately 100 μL of blood was collected into a serum capillary collection tube (Ram Scientific, Yonkers, NY) and transported back to the laboratory, where serum samples were separated by centrifugation. Serum was stored at −20°C in the field laboratory in Homabay until transported monthly to the main KEMRI NTD laboratory located in Kisumu. In Kisumu, samples were stored at −80°C until sent to a KEMRI laboratory in Nairobi, where samples were tested for antibody responses to a panel of antigens by MBA (described in the Muliplex bead assay section below).

### Treatment.

All eligible individuals in the community-wide treatment arm were offered annual treatment with single doses of PZQ (40 mg/kg) and albendazole (ALB) (400 mg) approximately 2 months after data collection. In the school-based treatment arm, the current WHO-recommended strategy to treat only SAC was followed.^3^ In both study arms, because no current guidelines exist for the inclusion of PSAC in schistosomiasis control programs, only PSAC identified as positive for *S. mansoni* infection by Kato-Katz were treated with crushed PZQ under the supervision of a medical professional. Coartem (artemether [20 mg/dose] and lumefantrine [120 mg/dose]) were provided to individuals with malaria. Treatment with iron supplementation was provided for persons with mild and moderate anemia, and individuals with severe anemia were referred to the subcounty hospital according to the Kenya National Clinical Guidelines for Nutritional and Hematologic Conditions.^23^

### Multiplex bead assay.

An MBA was used to analyze antibody responses to multiple antigens at one time from a single serum sample.^20^ The following schistosome antigens were included in the panel for our study: *S. mansoni* soluble egg antigen (SEA)^25^ and Sm25, an integral glycoprotein found in microsomal preparations of *S. mansoni* adult worms (GenBank Accession M37004.1).^26^,^27^ The *Sm25* gene was cloned into BD BaculoGold^TM^ pAcSecG2T Baculovirus Transfer Vector (BD 554797; Fisher Scientific, Waltham, MA), and the expressed Sm25 recombinant proteins from Sf-9 insect cells were purified using glutathione agarose beads. SEA was coupled to SeroMap microsphere beads (Luminex Corp., Austin, TX) in phosphate-buffered saline (PBS) at pH 7.2 using 120 μg protein for 12.5 × 10^6^ beads, and Sm25 was coupled in PBS buffer at pH 7.2 using 12 μg of protein/12.5 × 10^6^ beads as previously described.^28^ Test sera were diluted 1:400 in PBS buffer (pH 7.2) containing 0.3% Tween 20, 0.02% sodium azide, 0.5% casein, 0.5% polyvinyl alcohol, 0.8% polyvinylpyrrolidone, and 3 μg/mL *Escherichia coli* extract. Duplicate samples were tested as previously described.^28^,^29^ Samples having a coefficient of variation of > 15% between duplicate wells for > 3 positive antibody responses were repeated. Cutoff values of 713.5 median fluorescence intensity (MFI)-backgound (bg) units for SEA (sensitivity = 97.5%, specificity = 100%) and 52.5 MFI-bg units for Sm25 (sensitivity = 93.5%, specificity = 97.3%) were calculated at the Centers for Disease Control and Prevention (CDC) from receiver-operating characteristic curves using sera from 46 stool-positive *S. mansoni* patients, presumed negative sera from 65 adult U.S. citizens with no history of foreign travel, and presumed negative sera from 45 U.S. children. Cutoffs were adjusted for instrument differences between CDC and KEMRI using a 2-fold serial dilution of a strong positive serum pool to generate a standard curve. The adjusted cutoffs for the KEMRI instrument were 965 MFI—bg units for SEA and 38 MFI—bg units for Sm25. Additional antigens for malaria, *Strongyloides*, *Ascaris*, *Giardia*, tetanus, and diphtheria were included in the MBA. Results from the additional antigens will be described elsewhere.

### Data analysis.

Statistical analyses were performed in SAS software version 9.3 (SAS Institute Inc., Cary, NC) and used the 5% level of significance. Frequencies and proportions were compared using either the Rao–Scott χ^2^ statistic,^30^ which incorporates a design correction into the analyses; logistic regression with variance estimates by a Taylor series expansion^31^ to account for cluster sampling; or in two analyses of *S. mansoni* classification, a standard Pearson χ^2^ because a design effect could not be estimated. For logistic regression, odds ratios (ORs) and 95% confidence intervals (CIs) are reported. Unless otherwise stated, results in this article are for PSAC only and analyses are restricted to children with MBA results only.

## Results

A total of 4,611 PSAC were enrolled in the study between 2012 and 2014. Of those enrolled, serum samples were available from 3,612 (78.3%) children and were tested by MBA. Mean age of enrollment at baseline was 3.0 years, decreased slightly to 2.8 years in year 2 and remained at 2.8 years in year 3. In each year of the study, the youngest (1 year old) and oldest age (5 years old) groups were somewhat underrepresented. The distribution of PSAC in each age group is shown in Table 1. Malaria prevalence as determined by thick blood smear increased significantly from year 1 to 2 (OR = 1.80, CI = 1.03–3.16, *P* = 0.04) and remained elevated in year 3 (Table 2). Approximately one-third of all PSAC had anemia at baseline. Prevalence of anemia increased to 40.9% in year 2, resulting in increased odds of being anemic (OR = 1.35, CI = 1.08–1.68, *P* = 0.01) with approximately 32% of anemic children classified as having moderate to severe anemia (Table 2). Anemia was significantly associated with malaria (Rao–Scott χ^2^_(1)_ = 129.60, *P* < 0.001) and older age (Rao–Scott χ^2^_(4)_ = 109.09, *P* < 0.001) but was not associated with *S. mansoni* infection determined by either Kato-Katz (Rao–Scott χ^2^_(1)_ = 0.40, *P* = 0.53) or MBA (Rao–Scott χ^2^_(1)_ = 0.08, *P* = 0.77). The proportion of children with hematuria was high at baseline (33.8%) but was significantly lower in years 2 (1.5%, OR = 0.03, CI = 0.02–0.06, *P* < 0.001) and 3 (3.9%, OR = 0.08, CI = 0.04–0.15, *P* < 0.001; Table 2). None of the filtered urine samples was positive for *S. haematobium* eggs.

**Figure 1.**
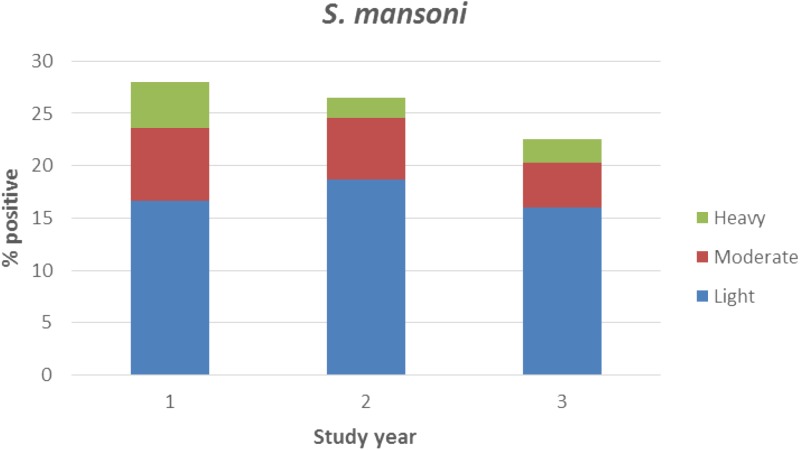
Prevalence and intensity of *Schistosoma mansoni* infection measured by Kato-Katz among preschool-aged children in each study year.

At baseline, the overall prevalence of *S. mansoni* infection by Kato-Katz was 28.0%, with 40.3% of infections classified as moderate or heavy intensity. *Schistosoma mansoni* infection significantly increased with age (Rao–Scott χ^2^_(4)_ = 58.69, *P* < 0.001). After one round of MDA in the study villages, there was minimal change in odds of *S. mansoni* infection among PSAC (OR = 0.93, CI = 0.70–1.23, *P* = 0.61), but there was a significant decrease in the percentage of moderate and heavy-intensity infections from 11.3% to 7.9% and the odds of moderate- or heavy-intensity infection (OR = 0.67, CI = 0.47–0.97, *P* = 0.03). By year 3, after two rounds of treatment, overall prevalence was still > 20% and nearly 30% of infections were classified as moderate or heavy intensity (Figure 1

). In contrast to *S. mansoni* infection, very low rates of STH infection were observed. Prevalence of any STH infection was < 3% in each study year (data not shown).

**Figure 2.**
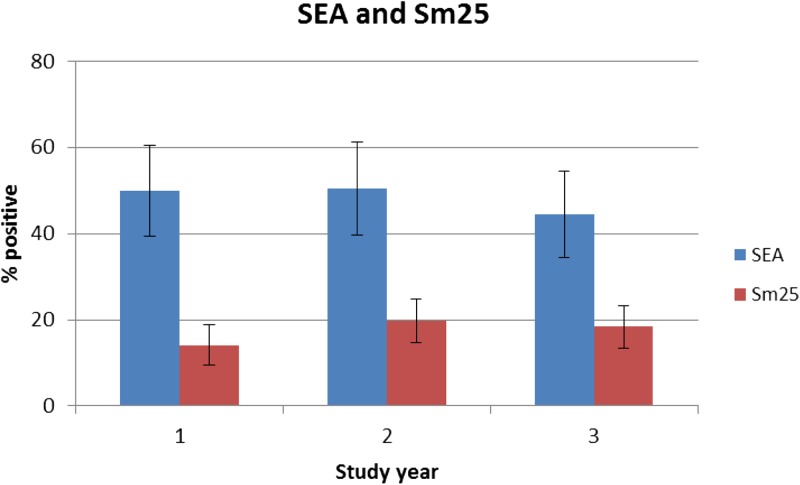
Prevalence of antibody responses to soluble egg antigen (SEA) and Sm25 by study year measured by multiplex bead assay.

**Figure 3.**
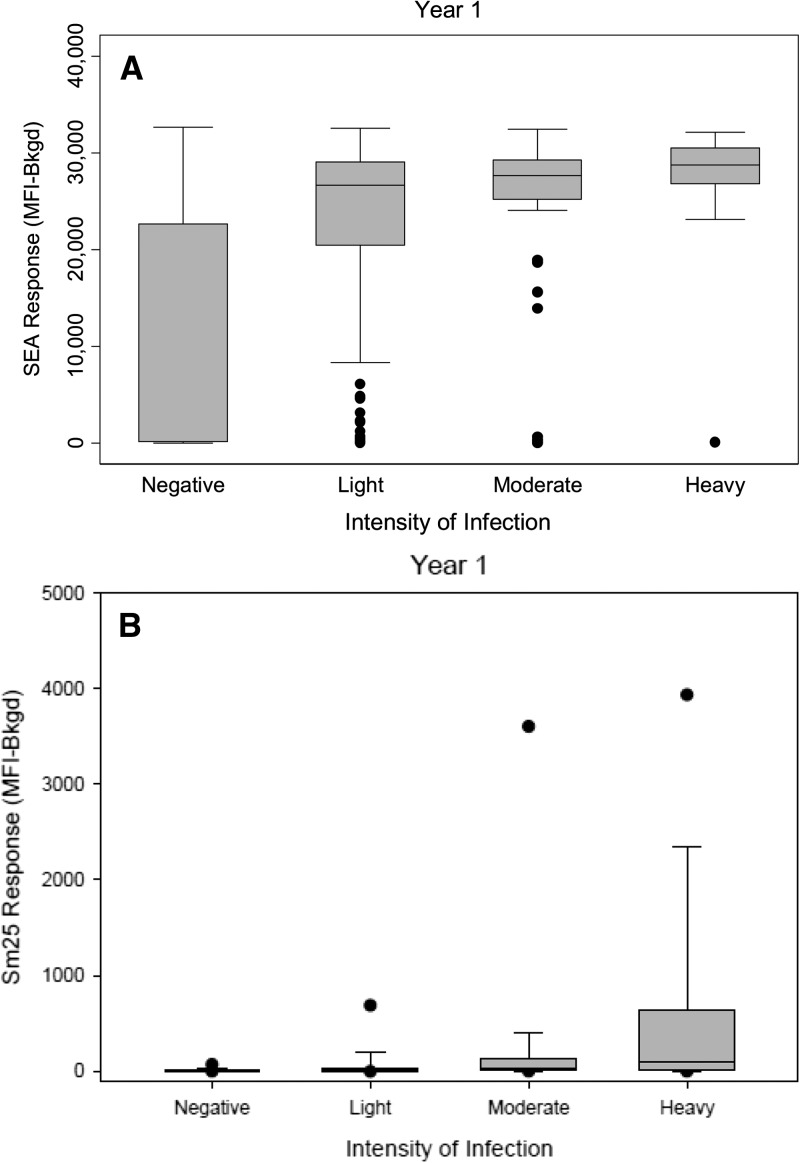
Antibody responses to (**A**) soluble egg antigen (SEA) and (**B**) Sm25 were significantly associated (*P* < 0.001) with intensity of infection measured by Kato-Katz. Boxes enclose 25th and 75th percentile. Lines inside the boxes represent median MFI values.

The overall prevalence of PSAC with antibody responses to SEA was high at baseline (50.0%) (Figure 2

). After one round of MDA, there was minimal change in odds of SEA positivity (OR = 1.02, CI = 0.79–1.32, *P* = 0.89). However, after two rounds of treatment, there was a slight decrease in odds of SEA positivity (OR = 0.80, CI = 0.63–1.02, *P* = 0.08). In contrast to the SEA results, prevalence of PSAC with antibodies to Sm25 was lowest at baseline (14.1%) and higher in years 2 (19.8%) and 3 (18.4%) (Figure 2). After one round of MDA, odds of Sm25 positivity increased significantly (OR = 1.51, CI = 1.14–2.02, *P* = 0.005) and remained significantly higher than baseline after two rounds of MDA (OR = 1.37, CI = 1.07–1.76, *P* = 0.01). There was a significant association between dichotomized anti-SEA antibody response and intensity of infection measured by Kato-Katz (Pearson χ^2^_(3)_ = 230.22, *P* < 0.001; Figure 3A

). Similarly, there was a significant association between dichotomized Sm25 responses and intensity of infection (Pearson χ^2^_(3)_ = 129.43, *P* < 0.001; Figure 3B).

**Figure 4.**
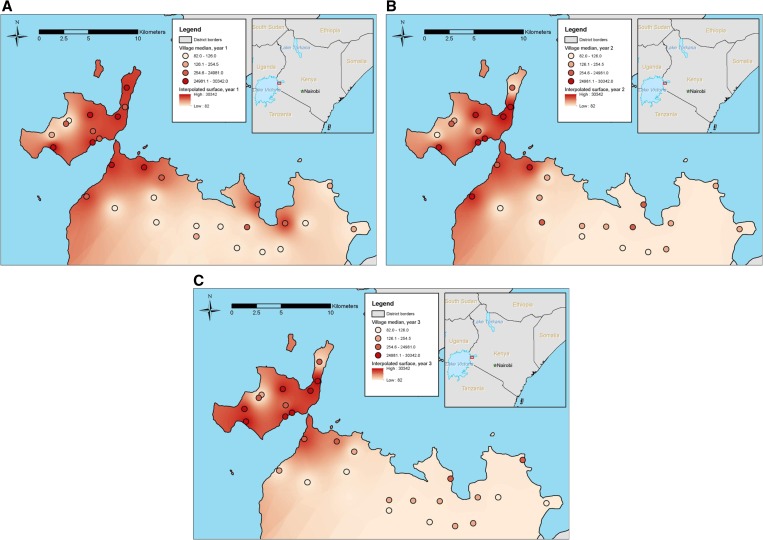
(**A**) Year 1: significant decrease (*P* = 0.004) in the odds of a positive soluble egg antigen (SEA) response was observed with each additional kilometer away from Lake Victoria. (**B**) Year 2: after one round of treatment, median SEA responses remained high on Rusinga Island, but decreased in some villages on the mainland closest to the lake. (**C**) Year 3: after two rounds of treatment, median SEA responses remained high on Rusinga Island, but continued to decrease in some villages on the mainland closest to the lake.

Although all of the study villages were located relatively close (< 5 km) to Lake Victoria, a gradient of antibody responses to SEA was observed. Median SEA responses for the villages at baseline ranged from 4 to 32,685 MFI. The highest responses were observed on the island of Rusinga in the northwest corner of the subcounty and there was a significant decrease in the odds of a positive SEA response with each additional kilometer away from the lake (OR = 0.22, CI = 0.08–0.62, *P* = 0.004, Figure 4A

) after controlling for study year. After each round of treatment, median SEA responses remained very high on Rusinga, but decreased in some villages on the mainland closest to the lake (Figure 4B and C). In any study year, there were no differences in stool results or antibody responses between the two study treatment arms.

**Figure 5.**
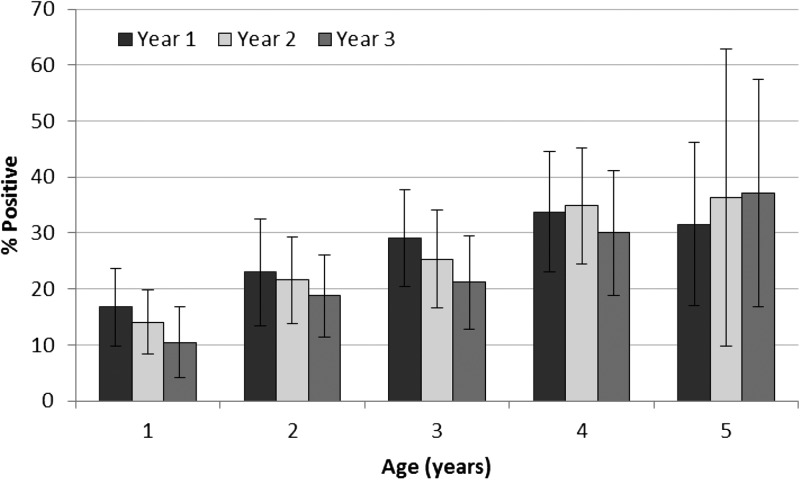
*Schistosoma mansoni* prevalence by age and study year measured by Kato-Katz.

**Figure 6.**
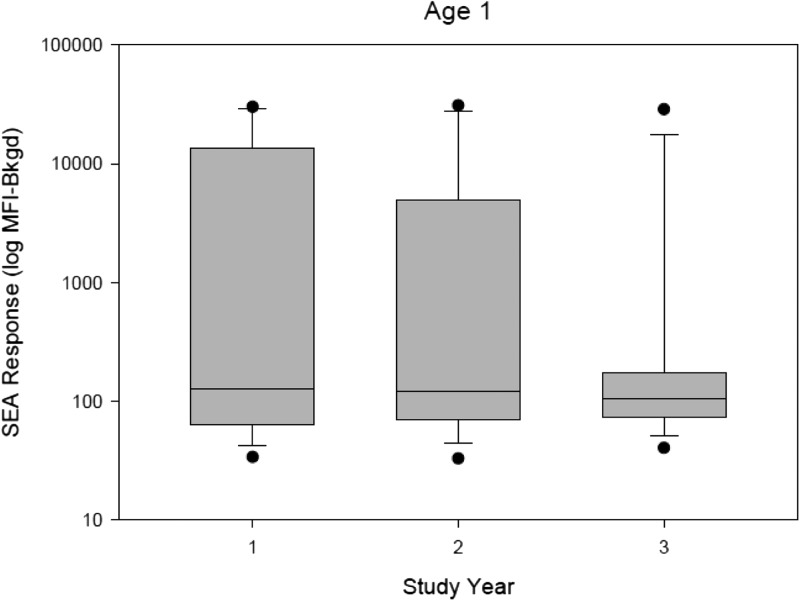
A significant reduction (*P* < 0.05) in the median soluble egg antigen (SEA) MFI values among 1-year olds after two rounds of mass drug administration was observed. Boxes enclose 25th and 75th percentile. Lines inside the boxes represent median MFI values.

There was no significant change in prevalence of *S. mansoni* infection by age after two rounds of treatment as determined by stool examination (all χ^2^_(2)_ < 3.11, *P* > 0.21), and prevalence was > 20% every year for older PSAC (Figure 5

). In contrast to the egg data, there was a decrease in the proportion of 1-year-olds with positive SEA responses from 33.1% in year 1 to 13.2% in year 3 (OR = 3.25, CI = 1.75–6.08, *P* < 0.001). Furthermore, there was a significant reduction in the median SEA MFI values among 1-year olds after two rounds of MDA (*P* < 0.05) (Figure 6

). The same reduction was not observed in any other age group.

## Discussion

In our prospective, cluster-randomized trial in Mbita subcounty, western Kenya, *S. mansoni* infection prevalence was high among a group of young children. Although our findings were consistent with previous reports of high rates of *S. mansoni* infection among SAC in this subcounty,^22^ this study provides unique data on PSAC. At baseline, using the relatively insensitive Kato-Katz method on a single stool sample, nearly 30% of PSAC were identified as infected with a parasite that is often considered to be of little public health importance in this age group. As expected, prevalence of *S. mansoni* infection as determined by stool examination increased with age. However, our results highlight that young children were being exposed to contaminated water early in life, in some cases up to 3 years or more before they would be eligible for inclusion in MDA programs. Our findings add to the growing body of evidence that children are at risk for schistosomiasis at a very early age.

Despite a high rate of *S. mansoni* infection in our study population, there was a relatively low prevalence of STH infection in the same group. Although the environmental conditions in Mbita subcounty were conducive to STH transmission and despite the lack of improved water, sanitation, and hygiene interventions, the prevalence of STH infection was much lower than expected. Before the study, there was one MDA for STH in 2009 and there have been anecdotal reports of unprogrammed deworming with ALB for STH. These factors may have contributed to the low prevalence of STH observed in our study.

Schistosomiasis control program strategies have traditionally aimed to reduce prevalence of moderate- and heavy-intensity infections. More recently, additional strategies are being considered to interrupt transmission. Attaining program goals are therefore dependent on diagnostic tools that can adequately measure prevalence and intensity of infection. Although it is commonly believed that lower intensity infections do not have significant impact on morbidity due to the disease, there is growing recognition that even light-intensity infections can have considerable impact on the health of children.^32^ In our study, after two rounds of MDA, traditional parasitologic methods showed little change in prevalence of *S. mansoni* infection in PSAC. There was a significant decrease in heavy-intensity infections after the first round of treatment in our study, but approximately 29% of infections were still classified as moderate or heavy-intensity after two rounds of MDA. Our results support the growing concern that a single annual round of MDA in high-prevalence areas may not be sufficient to achieve program goals.^33^

In our study, stool examinations were less sensitive than serology. It is possible that prevalence of *S. mansoni* infection was underestimated by only performing a single stool examination, but other studies have shown that in high-prevalence areas, multiple stool examinations conducted on consecutive days performed no better than a single examination.^8^ In complex- and resource-constrained program settings, it is not feasible to collect multiple stool samples over consecutive days. We aimed to provide information that could be compared with current programmatic approaches. In addition, it is unlikely that antibody results were significantly influenced by *S. haematobium* infection. Despite high rates of hematuria at baseline, there was no visible blood in any urine sample. High rates of hematuria were not observed in years 2 and 3. The use of different brands of urine dipsticks in years 1 and 2 may have impacted hematuria results, but the absence of *S. haematobium* eggs on urine filtration supports the claim that antibody results were likely attributable to *S. mansoni* infections. Although there have been reports of few isolated foci of *S. haematobium* in areas adjacent to Mbita,^34^ none have been identified in our study area. Furthermore, it is possible that cutoff values for the MBA were inaccurate, leading to incorrect prevalence estimates. The ability to define robust cutoffs for serological assays can be challenging and is often limited by the availability of well-characterized panels of samples to determine appropriate cutoffs. Despite potential limitations, it is clear that a high proportion of children in our study were exposed to *S. mansoni* at an early age. Our results showed good correlation between antibody responses to SEA and Sm25 and intensity of infection measured by Kato-Katz. In addition, we observed a significant association between antibody responses and distance to Lake Victoria. This inverse gradient relationship has been observed with stool results,^35^,^36^ but to our knowledge has not previously been documented by serology.

Although there are limitations to using serology to distinguish between present and past schistosome infections, longitudinal monitoring of antibody responses could provide useful information on possible changes in exposure and may provide an advantage over traditional parasitologic methods. In addition to parasitologic methods, schistosomiasis control programs often include morbidity markers such as anemia to assess program impact. However, these markers are often difficult to measure and are not unique to infection with *Schistosoma* spp. In our study, we observed high rates anemia that were associated with malaria and not *S. mansoni* infection. As control programs successfully implement interventions, reduced transmission of schistosomiasis will result in fewer infections and lower prevalence of infection-specific antibody in cohorts of young children. Malaria control programs have described the use of seroincidence among young children born after control measures have been put in place as a way to measure current and historical transmission within communities.^37^–^39^ Recently, in a lymphatic filariasis program setting, antibody responses were used to distinguish areas where programs had been implemented and successful, suboptimally implemented, and not implemented at all.^40^ This type of information would be useful for understanding how effective control programs have been. The use of serologic markers has most often been used in the context of low-prevalence settings or surveillance. However, our results showed a decline in antibody responses among young children in an area where transmission was clearly ongoing. Although further studies are needed to support this finding, these results provide preliminary evidence that program impact can be monitored using serologic responses.

Despite the limitations of this study, we believe the ability to use serologic assays to monitor schistosomiasis control programs could potentially provide advantages over the current stool-based approach. It is often easier to collect blood samples versus stool and an additional advantage is the ability to directly observe the collection of fingerstick blood, whereas the same opportunity does not exist for stool or urine collection. Furthermore, very small quantities of blood or dried bloodspots can be used in MBAs that can simultaneously test for a variety of diseases of public health importance at the same time. As there is often overlap of many of these diseases, this opens opportunities for integrated program monitoring, including vaccine coverage surveys. As schistosomiasis programs consider the feasibility of transitioning from control to elimination, the ability to document reduced seroincidence strengthens the evidence of elimination of transmission.

## Figures and Tables

**Table 1:** Age and sex distribution of preschool-aged children enrolled and tested by multiplex bead assay in each study year

Years (months)	Year											
	1 (baseline)				2				3			
*n*	%	Female	%	*n*	%	Female	%	*n*	%	Female	%
1 (12–23)	154	14.0	79	51.3	181	15.4	92	50.8	235	17.6	124	52.8
2 (24–35)	225	20.4	134	59.6	259	22.1	126	48.6	296	22.2	156	52.7
3 (36–47)	274	24.8	142	51.8	284	24.2	163	57.4	332	24.9	165	49.7
4 (48–59)	354	32.1	169	47.7	427	36.4	233	54.6	433	32.4	218	50.3
5 (60–71)	96	8.7	46	47.9	23	2.0	13	56.5	39	2.9	24	61.5

**Table 2:** Prevalence of malaria, anemia, and hematuria among preschool-aged children in each study year

	Year								
	1 (baseline)			2			3		
	*n*	Positive	%	*n*	Positive	%	*n*	Positive	%
Malaria	727	91	12.5	1,160	238	20.5	1,306	268	20.5
Anemia	1,096	371	33.9	1,170	478	40.9	1,323	517	39.1
Mild		250	67.4		345	72.2		351	67.9
Moderate		113	30.5		131	27.4		152	29.4
Severe		8	2.2		2	0.4		14	2.7
Hematuria	1,025	346	33.8	1,103	17	1.5	1,241	48	3.9
